# A Multigenic Network of ARGONAUTE4 Clade Members Controls Early Megaspore Formation in *Arabidopsis*

**DOI:** 10.1534/genetics.116.188151

**Published:** 2016-09-01

**Authors:** Elvira Hernández-Lagana, Daniel Rodríguez-Leal, Judith Lúa, Jean-Philippe Vielle-Calzada

**Affiliations:** Centro de Investigación y de Estudios Avanzados CINVESTAV, UGA Laboratorio Nacional de Genómica para la Biodiversidad, Grupo de Desarrollo Reproductivo y Apomixis, 36821 Irapuato, México

**Keywords:** ARGONAUTE, *Arabidopsis*, RdDM, gametogenesis, ovule

## Abstract

The development of gametophytes relies on the establishment of a haploid gametophytic generation that initiates with the specification of gametophytic precursors. The majority of flowering plants differentiate a single gametophytic precursor in the ovule: the megaspore mother cell. Here we show that, in addition to *argonaute9* (*ago9*), mutations in other *ARGONAUTE (AGO)* genes such as *ago4*, *ago6*, and *ago8*, also show abnormal configurations containing supernumerary gametophytic precursors in *Arabidopsis thaliana*. Double homozygous *ago4 ago9* individuals showed a suppressive effect on the frequency of ovules with multiple gametophytic precursors across three consecutive generations, indicating that genetic interactions result in compensatory mechanisms. Whereas overexpression of AGO6 in *ago9* and *ago4 ago9* confirms strong regulatory interactions among genes involved in RNA-directed DNA methylation, *AGO8* is overexpressed in premeiotic ovules of *ago4 ago9* individuals, suggesting that the regulation of this previously presumed pseudogene responds to the compensatory mechanism. The frequency of abnormal meiotic configurations found in *ago4 ago9* individuals is dependent on their parental genotype, revealing a transgenerational effect. Our results indicate that members of the *AGO4* clade cooperatively participate in preventing the abnormal specification of multiple premeiotic gametophytic precursors during early ovule development in *A. thaliana*.

THE alternation between the diploid-sporophytic and the haploid-gametophytic generation is a crucial distinction between the life cycle of plants and animals. Whereas in mammals and insects the cell lineage giving rise to gametes differentiates during embryogenesis (Bendel-Stenzel *et al.* 1998; Donoughe *et al.* 2014), in plants the gametogenic lineage initiates during floral development in the adult organism (Yadegari and Drews 2004). The onset of the female reproductive phase in most sexual flowering plants is defined by the specification of a single diploid megaspore mother cell (MMC) within the sporophytic nucellus, in the developing ovule (Bajon *et al.* 1999). Before gametogenesis, the MMC divides meiotically to produce four haploid megaspores. As in the majority of sexual species, in *Arabidopsis thaliana* (*Arabidopsis*) a single meiotically derived cell [the functional megaspore (FM)] differentiates prior to dividing mitotically and giving rise to the female gametophyte. The nucleus of the FM performs three mitotic divisions generating eight nuclei that subsequently undergo cellularization and differentiation to form accessory cells (the synergids and the antipodals), and two types of gametes: the egg cell and the central cell, which after fertilization will produce the embryo and the endosperm, respectively (Robinson-Beers *et al.* 1992; Reiser and Fischer 1993; Schneitz *et al.* 1995; Sheridan *et al.* 1999; Drews and Yadegari 2002).

Despite its prime importance for plant reproduction, the genetic basis and molecular mechanisms that control the somatic-to-reproductive transition are poorly understood. Only few mutants affecting specification of gamete precursors in the ovule have been found and characterized in different species. Mutants affecting the plant-specific MADS-box domain transcription factor *NOZZLE/SPOROCYTELESS* (*NZZ/SPL*) are defective in differentiating both microspore mother cells and MMCs (Yang *et al.* 1999). Similar to *nzz/spl*, the *Arabidopsis windhouse1* (*wih1*) and *windhouse2* (*wih2*) mutants are not able to properly differentiate an MMC; instead, nucellar cells acquire a parenchyma-like identity (Lieber *et al.* 2011). Other mutants promote the differentiation of several MMCs. In maize, for example, the loss of function of *MULTIPLE ARCHEOSPORES 1 (MAC1)*, a gene encoding a leucine-rich repeat receptor-like kinase protein (LRR-RLK), promotes the development of numerous MMCs that can undergo meiosis (Sheridan *et al.* 1999). A similar phenotype was found in the *multiple sporocyte 1* (*msp1*) mutant of rice, a gene encoding a LRR-RLK protein (Nonomura *et al.* 2003). In *Arabidopsis*, the differentiation of more than one female gamete precursor was first reported in mutants affecting small RNA (sRNA) pathways. Dominant *argonaute9 (ago9)*, *suppressor of gene silencing 3 (sgs3)*, *rna-dependent rna polymerase 2 (rdr2)*, *rna-dependent rna polymerase 6 (rdr6)*, *dicer-like 3 (dcl3)* mutants, and a double mutant defective in both *RNA POLYMERASE IV* and *POLYMERASE V* genes (*nrpd1a nrpd1b)*, show multiple gametophytic precursors in the premeiotic ovule. Furthermore, some of these ectopic cells are able to develop female gametophytes that bypass meiosis, a phenotype resembling aposporous mechanisms that prevail in some plant species reproducing by apomixis (Olmedo-Monfil *et al.* 2010). More recently, a similar phenotype was reported for a dominant mutant affected in *MNEME* (*MEM*), a gene encoding an RNA helicase of the DEAD-box family (Schmidt *et al.* 2011).

Epigenetic mechanisms involved in the development of the animal germline have also been reported (Hajkova *et al.* 2002; Loriot *et al.* 2003; Brennecke *et al.* 2007; Brennecke *et al.* 2008; Kelly 2014). For example, it is well known that differentiation of primordial germ cells encompasses extensive DNA demethylation and histone replacement (Seki *et al.* 2005; Leitch *et al.* 2013). Similarly, recent findings demonstrated that highly active chromatin changes occur during MMC specification in *Arabidopsis*, suggesting that a reorganization of the epigenetic landscape takes place during the somatic-to-reproductive transition (She *et al.* 2013). In plants, sRNAs are involved in epigenetic modifications through the RNA-dependent DNA methylation (RdDM) regulation pathway (Matzke and Mosher 2014). RdDM initiates with the transcription of chromatin-enriched loci by the plant-specific DNA-directed RNA Polymerase IV (Pol IV). Pol IV transcripts are converted into long double-stranded RNA molecules by the action of RDR2, and subsequently sliced by DCL3 into 24 nt sRNAs that are loaded by AGO proteins such as AGO4, AGO6, or AGO9. These proteins interact with nascent transcripts produced by Polymerase V (Pol V), promoting the recruitment of *de novo* DNA methyltransferases such as DOMAINS REARRANGED METHYLTRANSFERASE 2 (DRM2) as well as histone methyltransferases and chromatin remodelers, to ultimately cause the reinforcement of heterochromatin (Law and Jacobsen 2010; Simon and Meyers 2010; Rowley *et al.* 2011; Zhang and Zhu 2012; Zhong *et al.* 2014). AGOs are a class of PAZ/PIWI domain-containing proteins that have undergone a high degree of gene duplication in plants (Vaucheret 2008; Zhai *et al.* 2014). In *Arabidopsis*, a phylogenetic analysis defined three distinct clades: the AGO1/5/10, the AGO2/3/7, and the so-called AGO4 clade composed of AGO4, AGO6, AGO8, and AGO9 (Supplemental Material, Figure S1; Vaucheret 2008). To date, only AGO4, AGO6, and AGO9 are shown to bind heterochromatic small interfering RNAs (siRNAs) that mostly target repetitive genomic regions and transposable elements (TE) (Zilberman *et al.* 2004; Zheng *et al.* 2007, 2013; Duran-Figueroa and Vielle-Calzada 2010; Havecker *et al.* 2010; Olmedo-Monfil *et al.* 2010; Eun *et al.* 2011). By contrast, *AGO8* is generally considered to be a pseudogene, mainly because a computational analysis predicted that its presumed coding sequence contains splicing-inducing frame shifts, suggesting the formation of a nonfunctional protein (Takeda *et al.* 2008).

Here we report that mutations in *AGO4*, *AGO6*, or *AGO8* lead to abnormal premeiotic ovules harboring more than one female gametophytic precursor, a phenotype reminiscent of defects found in *ago9* mutants. We also show that individuals defective in both *AGO4* and *AGO9* show a suppressive effect on the frequency of ovules harboring this phenotype, revealing a genetic interaction between these two genes that leads to compensatory mechanisms in the control of cell specification. A detailed genetic and cytological analysis indicates that the frequency of premeiotic ovules showing ectopic cells is influenced by parental genotypes involving the function of AGO proteins other than AGO4 and AGO9. Gene expression analysis and *in situ* protein immunolocalization indicate that *AGO6* is overexpressed in *ago9* but not in *ago4* or *ago4 ago9* ovules, suggesting the existence of an interaction between AGO6 and AGO9 that partially depends on the activity of AGO4. By contrast, *AGO8* is only overexpressed in *ago4 ago9* individuals, suggesting its possible role in the compensatory effect that contributes to restrict gametophytic cell fate. Our results reveal a multigenic network of interactions involving members of the AGO4 clade to control early megaspore formation, opening new possibilities for elucidating the canalized mechanisms that ensure the initial stages of sexual reproduction in *Arabidopsis*.

## Materials and Methods

### Plant material and growth conditions

The *ago4-6* (SALK_071772; Strickler *et al.* 2013), *ago5-4* (SALK_050483; Tucker *et al.* 2012), *ago6-2* (SALK_031553; Zheng *et al.* 2007), *ago8-1* (Havecker *et al.* 2012), *ago8-2* (SALK_010058), and *ago9-3* (SAIL_34_G10; Olmedo-Monfil *et al.* 2010) mutant alleles are in the Columbia (*Col*) background; whereas *ago1-37* (Yang *et al.* 2006) and *ago4-1* (Zilberman *et al.* 2003) are in the Landsberg *erecta* (L*er*) background. Both *Col* and L*er* wild-type plants were used as controls in the quantitative analysis of single mutant genotypes, whereas *Col* × L*er* F_1_ individuals were used as controls in the analysis of double mutant genotypes. Seeds were surface sterilized with chlorine gas and germinated under long-day conditions (16 hr light/8 hr dark) in MS medium at 22°. Seedlings were grown under greenhouse or growth chamber conditions (24°). Primer pairs used for genotyping are listed in Table S2.

### Cytological analysis of ovule development

For cytological examination of premeiotic ovules, gynoecia of 0.4–0.6 mm in length from wild-type and mutant plants were harvested and fixed in formalin-acetic acid-alcohol solution (40% formaldehyde, glacial acetic acid, 50% ethanol; in a 5:5:90 volume ratio) for 24 hr at room temperature. After fixation, samples were washed five times with absolute ethanol and stored in 70% ethanol at room temperature for 24 hr. Fixed gynoecia were dissected with hypodermic needles (1 mm insulin syringes), cleared in Herr’s solution (phenol:chloral hydrate:85% lactic acid:xylene:clove oil in a 1:1:1:0.5:1 proportion), and observed by differential interference contrast microscopy using a Leica DMR microscope.

### Quantitative real-time PCR

Total RNA from gynoecia bearing premeiotic ovules (0.5–0.6 mm in length) was isolated using Trizol (Invitrogen, Carlsbad, CA). Complementary DNA (cDNA) was synthesized from 1 µg of total RNA, using oligo dT and SuperScript reverse transcriptase II (Invitrogen). Primers for PCR were designed using the online program Primer 3 (v.0.4.0) and verified with OligoEvaluator (Sigma Chemical, St. Louis, MO) to discard dimer structure formation. PCR efficiencies of the target and reference genes were determined by generating standard curves, based on serial dilutions prepared from cDNA templates. PCR efficiency was calculated according to the slope of the standard curve (primers with 100% efficiency, the fold equals to 2). Each quantitative real-time PCR (qPCR) reaction was performed in a 10-µl volume consisting of 5 µl of 2× SYBR Green PCR Reaction Mix (Applied Biosystems, Foster City, CA), 3.5 µl of DNA template (10 ng/µl), 0.5 µl of forward primer (5 µM), 0.5 µl of reverse primer (5 µM), and 0.5 µl of ultrapure water. The qPCR reactions were performed using the StepOne Applied Biosystems and the data were analyzed using the StepOne software v2.2.2. The thermal profile consisted of 10 min at 95°, 40 cycles of 15 sec at 95°, and 1 min at 60°. Amplification results were collected at the end of the extension step. Primer sequences used for qPCR amplifications are listed in Table S2 and Table S4. A comparative 2^−ΔΔCt^ method was used for determining a relative target quantity of gene expression, and *ACTIN2* was used as a control (Czechowski *et al.* 2004). Reproducibility of the results was evaluated for each sample by running three technical and three biological replicates of each of the reactions and each genotype.

### Whole-mount immunolocalization

Whole-mount immunolocalization was performed as described in Escobar-Guzmán *et al.* (2015), with minor modifications. Gynoecia of 0.6 mm were harvested and fixed in paraformaldehyde (1× PBS, 4% paraformaldehyde, 2% triton), for 2 hr under continuous agitation on ice. After fixation, the samples were washed three times in 1× PBS and embedded in a matrix of 15% acrylamide:bysacrilamide (29:1) over positively charged slides (ProbeOn Plus; Fisher Scientific, Pittsburgh, PA) previously treated with Poly-L-lysine. After embedding, the samples were digested in an enzymatic cocktail (1% driselase, 0.5% cellulose, 1% pectolyase) in 1× PBS for 1 hr at 37°. Then, the samples were permeabilized for 2 hr in 1× PBS:2% triton and blocked by incubating them with 1% BSA (Hoffman La Roche, Nutley, NJ) for 1 hr at 37°. Incubation with AGO6 (Havecker *et al.* 2010) primary antibody was carried out overnight at 4° at a dilution of 1:100. Samples were washed for 8 hr in 1× PBS:0.2% triton, refreshing the solution each 2 hr. Subsequently, samples were incubated overnight with the secondary antibody Alexa Fluor 488 (Molecular Probes, Eugene, OR) at a dilution of 1:300. After a washing step of 8 hr, the samples were incubated with propidium iodide (500 µg/ml) in 1× PBS for 20 min and washed in 1× PBS for 40 min. Finally, samples were mounted in PROLONG (Molecular Probes). Sections of premeiotic ovules were captured on a laser scanning confocal microscope (LSM 510 META; Carl Zeiss, Thornwood, NY) with multitrack configuration for detecting propidium iodide [excitation with diode pump solid state (DPSS) laser at 568 nm, emission collected using band pass filter (BP) 575–615 nm] and Alexa 488 (excitation with Argon laser at 488 nm, emission collected using band pass filter (BP) 500–550 nm). Laser intensity and gain were equivalently set for all samples.

### Data availability

Sequence data from this article can be found in the European Molecular Biology Laboratory /GenBank data libraries under accession numbers At2g27040, At2g32940, At5g21030, and At5g21150. Additional mutant strains are available upon request. Table S2 contains names of primers used for genotyping and qPCR assays.

## Results

### All members of the *AGO4* clade are involved in the specification of female gametophytic precursors

**Figure 1:**
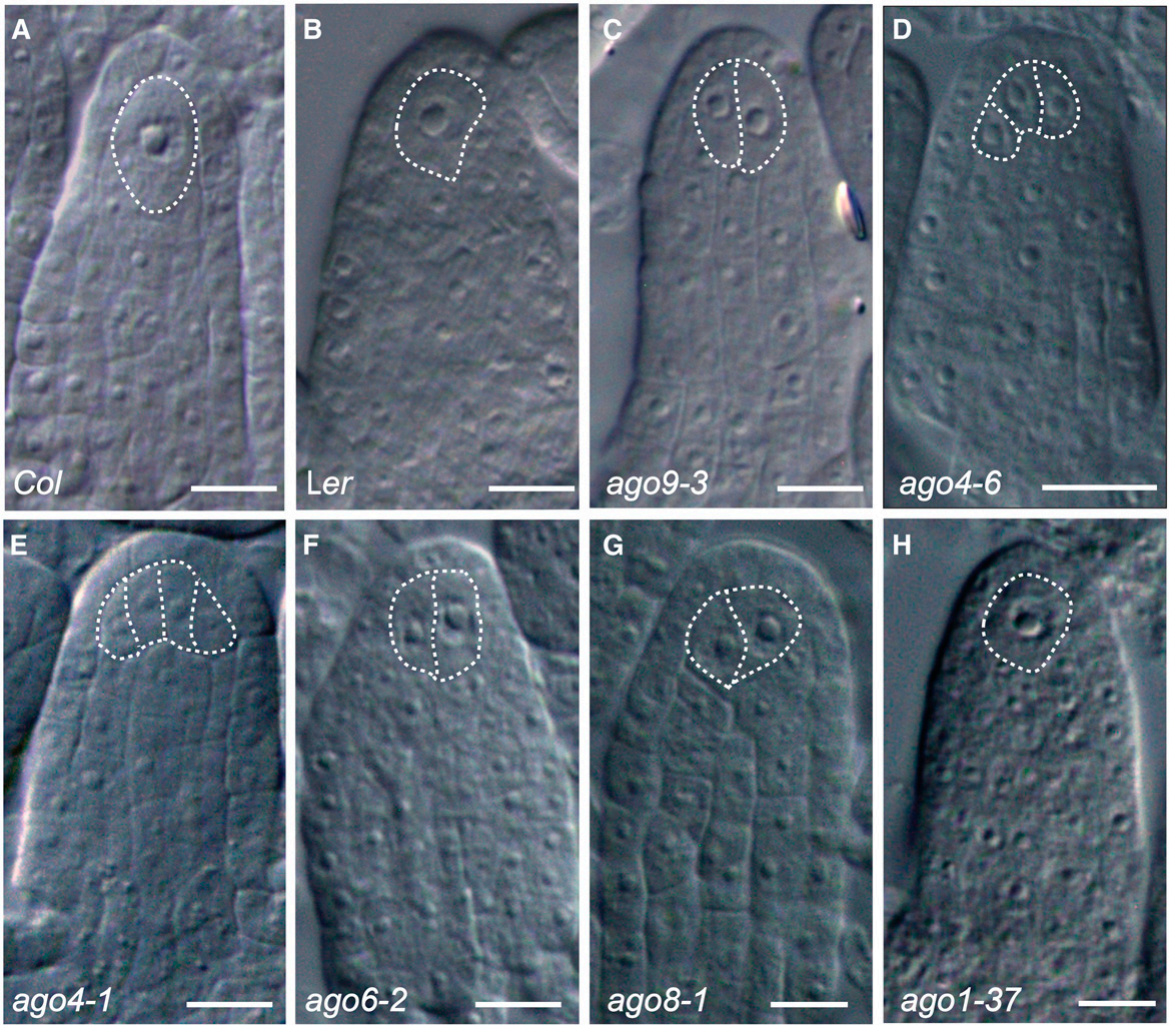
Phenotypic characterization of stage 1 ovules in wild type and *ago* mutants. (A) Wild-type ovule of *Col* showing a single MMC (class I). (B) Wild-type ovule of L*er* showing a single MMC (class I). (C) *ago9-3* ovule showing two enlarged cells (class II), (D) *ago4-6* ovule showing three abnormal enlarged cells (class III), (E) *ago4-1* ovule showing four enlarged cells (F) *ago6-2* ovule showing two enlarged cells (class III), (G) *ago8-1* ovule showing two enlarged cells (class II), and (H) *ago1-37* ovule showing a single MMC (class I). Bar, 20 µm.

It was previously reported that mutations in *AGO9* promote the development of more than one premeiotic gametophytic precursor during early ovule development (Olmedo-Monfil *et al.* 2010). The incomplete penetrance of this defect led us to suspect that additional factors could act redundantly to restrict cell specification in the ovule. To elucidate if close relatives of *AGO9* could have a role in the somatic-to-reproductive transition, we cytologically characterized early ovule development in mutant plants defective in *AGO4*, *AGO6*, and *AGO8*. We started by conducting a quantitative characterization of stage 1 ovules following the classification reported by Rodríguez-Leal *et al.* (Rodríguez-Leal *et al.* 2015). Stage 1 corresponds to premeiotic ovule primordia having a well-defined proximal-distal axis and absence of integument initiation. For this type of ovule, we defined three phenotypic classes based on the number of enlarged subepidermal cells reminiscent of gametophytic precursors corresponding to the MMC: class I includes ovules with a single gametophytic precursor, which by its subepidermal position corresponds to the MMC (Figure 1, A and B); class II corresponds to premeiotic ovules with two gametophytic precursors resembling twin MMCs (Figure 1C); and class III corresponds to premeiotic ovules containing more than two cells resembling the MMC (Figure 1, D and E). Under greenhouse conditions, 9.8% (*n* = 934) and 17.2% (*n* = 429) of *Col* and L*er* ovules showed two MMCs, respectively (Table 1), confirming that *Arabidopsis* ecotypes exhibit naturally occurring variation in the number of ovules with ectopic gametophytic precursors (Rodríguez-Leal *et al.* 2015). In contrast to wild type, *ago9-3* behaved as a dominant mutation showing 20.68% ± 2.18 (*n* = 385) and 28.93% ± 3.36 (*n* = 508) class II and class III ovules in heterozygous and homozygous mutant individuals, respectively.

**Table 1:** Phenotypic analysis of wild-type and mutant ovules at stage 1

Genotype	*n**^a^*	Class I*^b^*	Class II*^c^*	Class III*^d^*	Class II and III	AI*^e^*
*Col*	934	89.5 ± 1.23	10.49 ± 1.23	0	10.49 ± 1.23	3
L*er*	429	82.9 ± 1.61	16.52 ± 1.39	0.56 ± 0.33	17.24 ± 1.62	3
*ago1-37 (*L*er)*	369	86.90 ± 0.47	11.81 ± 0.83	0.97 ± 1.02	12.8 ± 0.54	3
*ago5-1 (Col)*	375	92.3 ± 0.55	7.59 ± 0.41	0	7.59 ± 0.41	3
*ago9-3/+* (*Col*)	385	78.48 ± 2.63	20.68 ± 2.18	0.82 ± 0.47	21.50 ± 2.63	4
*ago9-3* (*Col*)	508	71.05 ± 3.36	28.69 ± 3.36	0.32 ± 0.22	28.93 ± 3.36	4
*ago4-1/+* (L*er*)	270	79.77 ± 1.47	18.20 ± 0.94	2.00 ± 0.62	20.21 ± 1.47	3
*ago4-1* (L*er*)	276	61.58 ± 0.56	37.32 ± 0.90	1.07 ± 0.34	38.40 ± 0.56	4
*ago4-6/+* (*Col*)	649	83.16 ± 0.61	14.43 ± 0.43	2.39 ± 0.31	16.82 ± 0.61	3
*ago4-6* (*Col*)	356	66.54 ± 2.57	27.74 ± 0.25	5.70 ± 0.24	33.44 ± 1.48	3
*ago6-2/+* (*Col*)	426	87.69 ± 1.94	11.53 ± 1.99	0.75 ± 0.05	13.54 ± 1.85	3
*ago6-2* (*Col*)	220	74.31 ± 3.58	18.10 ± 2.18	7.56 ± 2.00	25.66 ± 3.58	4
*ago8-1/+* (*Col*)	334	87.50 ± 0.52	12.24 ± 0.41	0.24 ± 0.41	12.48 ± 0.52	3
*ago8-1* (*Col*)	1023	71.97 ± 3.28	26.11 ± 3.10	1.73 ± 0.71	27.84 ± 3.23	6
*ago8-2 (Col)*	456	70.29 ± 1.62	24.77 ± 1.67	4.91 ± 1.67	29.69 ± 1.62	5

Values are given as a percentage of the total number of ovules analyzed.

a Total of ovules analyzed.

b Ovules with a single MMC.

c Ovules with two enlarged MMC-like cells.

d Ovules with more than two enlarged MMC-like cells.

e Number of individuals included in the analysis.

Heterozygous *ago4-1/+* and *ago4-6/+* mutant individuals also showed abnormal numbers of ovules with gametophytic precursors, suggesting that these mutants are also dominant for the phenotype analyzed. The frequency of class II and class III ovules in heterozygous *ago4-1/+* (L*er* background) and *ago4-6/+* (Col background) individuals was 20% (*n* = 270) and 16.79% (*n* = 649), respectively (Table 1). As expected, homozygous *ago4-1* and *ago4-6* plants exhibited larger frequencies of class II and class III ovules compared to heterozygous plants (38.4 ± 0.56 and 33.44 ± 1.48, respectively; Table 1). Because past studies have shown that mutations in *AGO4* act as recessive loss-of-function alleles that can be rescued by hemizygous complementation (Zilberman *et al.* 2003), we conducted an additional cytological analysis in a randomly selected group of F_2_ and F_3_ individuals segregating for *ago4-6*, scoring a total of eight individuals per genotype at each generation. Whereas wild-type F_2_ plants that inherited two wild-type copies of *AGO4* showed a frequency of abnormal class II and class III ovules equivalent to wild-type Col (average of 10.65% ± 0.96; Table S1), F_2_ heterozygous *ago4-6/+* individuals showed a frequency of class II and class III ovules ranging between 28.84 and 37.71% (average of 31.82% ± 1.2; Table S1), which is similar to the frequency obtained for F_2_ homozygous *ago4/6* individuals (average of 33.23 ± 0,98; Table S1). Similar frequencies were obtained for segregating individuals of the F_3_ population resulting from self-pollination of a F_2_ heterozygous individual (Table S2), confirming that mutations in *AGO4* consistently cause dominant defects during megasporogenesis (Table S3).

In addition, heterozygous *ago6-2/+* individuals showed 13.54% (*n* = 426) of ovules harboring more than one gametophytic precursor (Table 1). Homozygous *ago6-2* plants exhibited the same phenotype at 25.66% (*n* = 220; Figure 1F, Table 1), indicating that *AGO6* also has a role in restricting the number of gametophytic precursors specified in the ovule primordium. Similar to homozygous *ago4-6*, homozygous *ago6-2* individuals showed a higher frequency of class III ovules than homozygous *ago9-3* individuals (Table 1). Finally, heterozygous *ago8-1/+* plants showed 12.48% (*n* = 334) of stage1 ovules harboring ectopic gametophytic precursors, whereas homozygous *ago8-1* and *ago8-2* individuals showed 27.84% (*n* = 1023) and 29.69% (*n* = 456) respectively (Figure 1G); indicating that *AGO8* is also involved in the restriction of cell specification, despite being previously reported as a pseudogene (Takeda *et al.* 2008). To determine if mutations in AGO genes not belonging to the AGO4 clade could also show defects in female gametophytic precursor specification, we analyzed *ago1-37*, a weak allele of *AGO1*, the main protein involved in microRNA-dependent regulatory pathways (Yang *et al.* 2006); and *ago5-1*, which had been previously shown to be involved in the promotion of mitosis during female gametogenesis in *Arabidopsis* (Tucker *et al.* 2012). Despite strong vegetative defects exhibited by *ago1-37* at almost all developmental stages (Yang *et al.* 2006), the frequency of ovules with ectopic gametophytic precursors in homozygous *ago1-37* and *ago5-1* individuals was no different from wild type (Figure 1H, Table 1). We therefore conclude that it is specifically the *AGO4* clade that plays an important role in restricting the number of female gametophytic precursors in the developing ovule.

### Genetic interactions between *AGO4* and *AGO9* affect the specification of female gametophytic precursors

**Figure 2:**
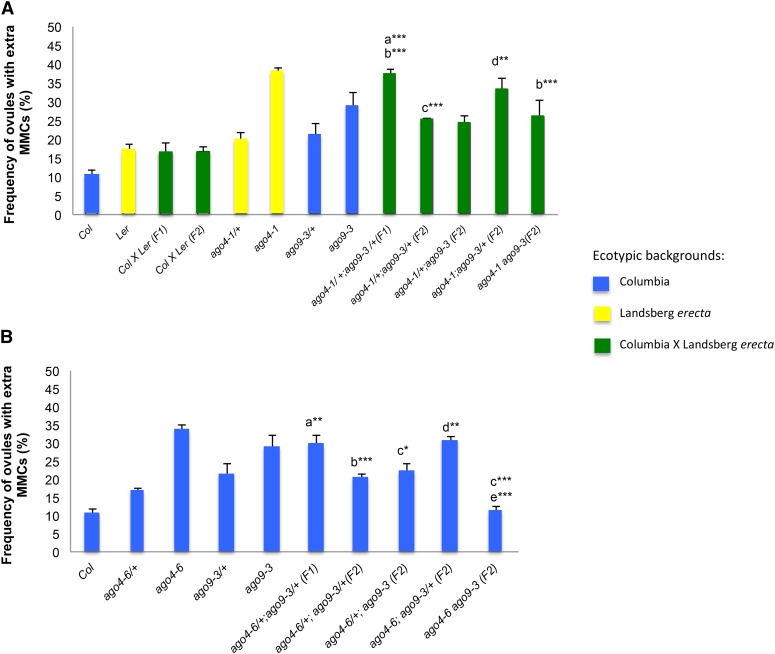
Genetic interactions between *ago4* and *ago9* during female gametophytic cell specification. (A) Quantitative analysis of stage 1 ovules showing more than one gametophytic precursor in genotypes of single and double *ago9-3 (Col) ago4-1* (L*er*) mutant individuals. Letters indicate pairwise results of two-tailed Fisher’s exact tests used to estimate statistical significance of possible differences between genotypes: a, comparison to *ago9-3/+*; b, comparison to *ago4-1/+*; c, comparison to *ago4-1/+;ago9-3/+* (F_1_); d, comparison to *ago9-3*;*ago4-1/+* (F_2_). * *P* < 0.05, ** *P* < 0.01, *** *P* < 0.001. (B) Quantitative analysis of stage 1 ovules showing more than one gametophytic precursor in genotypes of single and double *ago9-3* (*Col*) *ago4-6* (*Col*) mutant individuals. Letters indicate pairwise results of two-tailed Fisher’s exact tests used to estimate statistical significance of possible differences between genotypes: a, comparison to *ago4-6/+*; b, comparison to *ago4-6/+*;*ago9-3/+* (F_1_); c, comparison to *ago9-3*; d, comparison to *ago4-6/+*;*ago9-3* (F_2_); e, comparison to homozygous *ago4-6*. * *P* < 0.05, ** *P* < 0.01, *** *P* < 0.001. SDs were calculated on the basis of biological replicates for each genotype.

To determine possible genetic interactions between *AGO4* and *AGO9*, we conducted a phenotypic analysis in different genotypes involving the double mutant *ago4 ago9*. Homozygous *ago4-1* individuals show reduced DNA methylation primarily at CHG and CHH methylation sites (where H = A, T, or C) and less frequently at CG sites (Zilberman *et al.* 2003; Stroud *et al.* 2013). We crossed *ago4-1* to *ago9-3* individuals and produced plants segregating for both mutations. F_1_ double heterozygous ago4-1/+;ago9-3/+ individuals showed a frequency of ovules with ectopic gametophytic precursors reflecting an additive effect between these two mutants (Figure 2A). To analyze additional genotypic combinations, F_2_ individuals segregating for both mutations were also cytologically quantified. Interestingly, the frequency of ovules showing ectopic gametophytic precursors in F_2_ *ago4-1*/*+;ago9-3*/*+* was significantly lower than those expected under additive effects, and similar to frequencies observed in heterozygous plants for each single mutant (Figure 2A). Furthermore, F_2_ *ago4-1/+;ago9-3/ago9-3* plants exhibited a slightly lower frequency of abnormal ovules compared to single homozygous *ago9-3* individuals (Figure 2A), indicating that heterozygosity of *ago4-1* in the homozygous *ago9-3* background promotes a suppression of the mutant phenotype. On the other hand, F_2_ *ago4-1/ago4-1*;a*go9-3/+* individuals showed a frequency of ectopic gametophytic precursors lower than single homozygous *ago4-1* but higher than F_2_ *ago4-1/+;ago9-3/ago9-3* plants (Figure 2A). In addition, F_2_ double homozygous *ago4-1 ago9-3* individuals showed a significantly lower frequency of ovules with ectopic gametophytic precursors than homozygous *ago4-1* or *ago9-3* single mutant individuals (Figure 2A). These results indicate that the simultaneous absence of *AGO4* and *AGO9* tends to repress the differentiation of ectopic gametophytic precursors in the ovule. This repressive effect is less severe in F_2_ *ago4-1/ago4-1;ago9-3/+* plants, suggesting that a functional allele of *AGO9* in a homozygous *ago4* background tends to exacerbate the mutant phenotype. Strikingly, the frequency of ovules harboring multiple gamete precursors in F_2_ *ago4-1/+;ago9-3/+*, *ago4-1/+;ago9-3/ago9-3*, and *ago4-1/ago4-1;ago9-3/ago9-3* was similar, suggesting that additional genetic factors must contribute to restrict the specification of gametophytic precursors in the premeiotic ovule.

To determine if the ecotypic background could influence interactions between *AGO4* and *AGO9*, we conducted the same phenotypic analysis in *ago4-6*, a mutation generated in the *Col* and not the L*er* ecotype. As in the case of interactions between *ago4-1* and *ago9-3*, the frequency of abnormal stage 1 ovules was higher in F_1_ *ago4-6/+;ago9-3/+* individuals than in single heterozygous *ago4-6/+* or *ago9-3/+* mutants (Figure 2B). Also, F_2_ *ago4-6/+*; *ago9-3/+* individuals exhibited a lower frequency of abnormal ovules than F_1_ *ago4-6/+;ago9-3/+* plants (Figure 2B), confirming the results obtained with *ago4-1*. In addition, the suppressive effect observed in double homozygous individuals was stronger in *ago4-6 ago9-3* than in *ago4-1 ago9-3* individuals (Figure 2B), suggesting that the L*er* background exerts a stronger ecotypic effect than *Col* over the restriction of female gametophytic precursors. Overall, these results suggest that *AGO9* and *AGO4* genetically interact to restrict the differentiation of additional premeiotic precursors in the ovule, but also that additional genetic factors participate in this developmental process.

### Genetic interactions between *AGO4* and *AGO9* are influenced by parental genotypes

**Figure 3:**
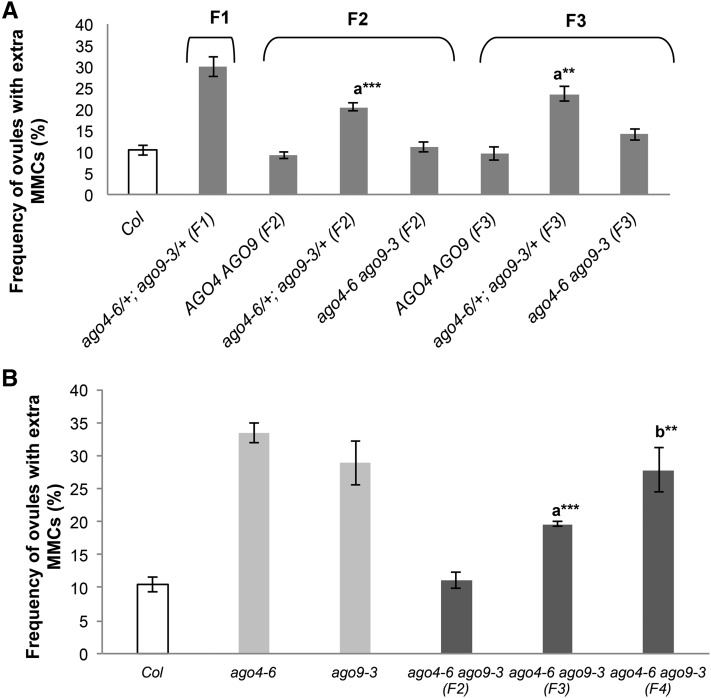
The number of female gametophytic precursors is influenced by the parental genotype. (A) Quantitative analysis of stage 1 ovules showing more than one gametophytic precursor in F_2_ and F_3_ segregant populations derived from *ago4-6/+ (Col)*; *ago9-3/+ (Col)* individuals. The letter indicates pairwise results of two-tailed Fisher’s exact tests used to estimate statistical significance of differences between genotypes: a, comparison to *ago4-6/+* ; *ago9-3/+* (F_1_). ** *P* < 0.01, *** *P* < 0.001. (B) The compensatory effect resulting from simultaneous loss of function of *AGO4* and *AGO9* is progressively diminished through consecutive generations. Letters indicate pairwise results of two-tailed Fisher’s exact tests used to estimate statistical significance of differences between genotypes: a, comparison to *ago4-6 ago9-3* (F_2_); b, comparison to *ago4-6 ago9-3* (F_3_). ** *P* < 0.01, *** *P* < 0.001. SDs were calculated on the basis of biological replicates for each genotype.

Emerging evidence suggests that mutations affecting epigenetic pathways can originate transgenerational, stable, epigenetic modifications (Reinders and Paszkowski 2009; Johannes and Colome-Tatche 2011; Mari-Ordonez *et al.* 2013). To test if this type of epigenetic effect could influence the function of *AGO4* or *AGO9*, we quantified the frequency of premeiotic stage 1 gametophytic precursors in the progeny of double heterozygous individuals segregating for mutations in these two genes. Equivalent frequencies were found in F_2_ and F_3_ wild-type segregant individuals (Figure 3A), discarding the possibility of strict transgenerational epigenetic effects and indicating that any abnormal frequency is caused by loss-of-function alleles for any of the two genes. We also analyzed stage 1 ovules in homozygous and heterozygous progeny of double heterozygous individuals. F_3_ double heterozygous plants exhibited a similar frequency of ectopic gametophytic precursors than F_2_ double heterozygous individuals, and double homozygous F_3_ individuals exhibited a frequency equivalent to double homozygous F_2_ individuals (Figure 3A). By contrast, the frequency of stage 1 ovules harboring ectopic gametophytic precursors increased progressively in F_3_ and F_4_ progeny originating from double homozygous plants (Figure 3B), indicating that the suppressive effect previously described is mitigated if not suppressed in progeny from double homozygous plants. These results indicate that the role of AGO4 and AGO9 in premeiotic gametophytic specification is influenced by the parental genotype, suggesting that loss of function of both genes triggers a compensatory effect that prevents the differentiation of ectopic cells in the developing ovule.

### *AGO6* and *AGO8* influence gametophytic specification in *ago4* and *ago9* mutant backgrounds

**Figure 4:**
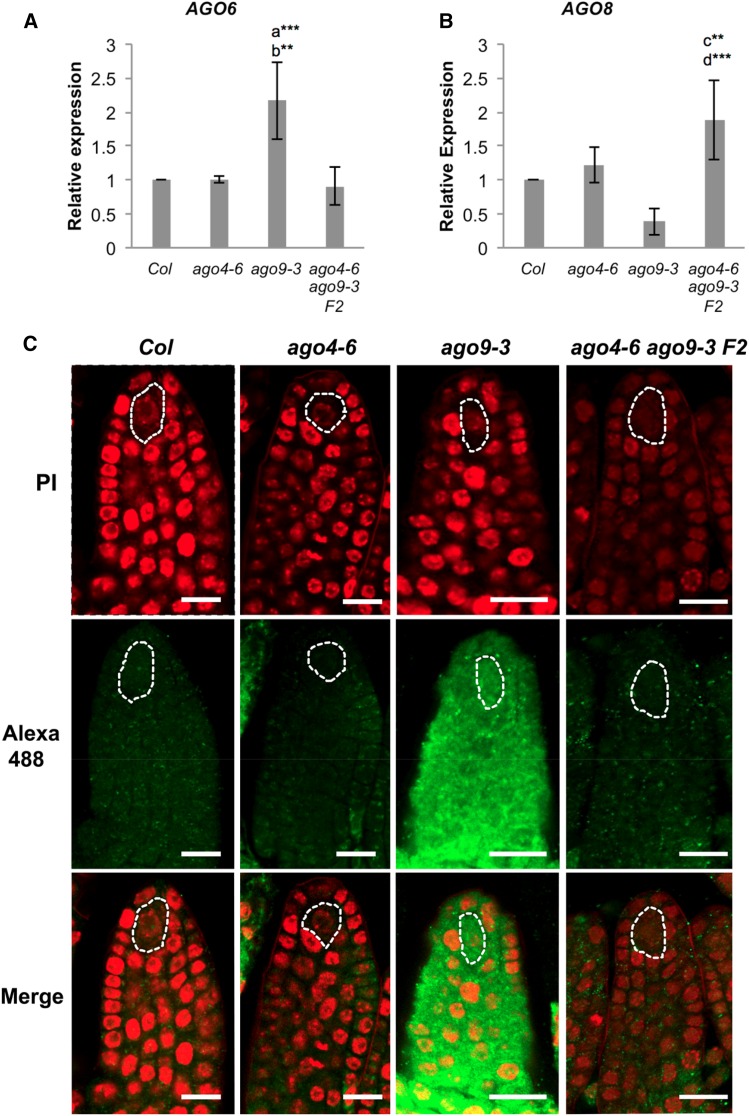
The expression of *AGO6* and *AGO8* is affected by loss-of-function mutations in *ago4* or *ago9*. (A) Expression of *AGO6* in developing gynoecia containing premeiotic ovules. (B) Expression of *AGO8* in developing gynoecia containing premeiotic ovules. The comparative 2^−ΔΔCt^ method was used for determining the relative level of gene expression as compared to wild type, using *ACTIN2* as internal control (Czechowski *et al.* 2004). Each histogram represents the mean of three biological replicates and shows the corresponding SD. Letters indicate pairwise results of two-tailed Fisher’s exact tests used to estimate statistical significance of possible differences between genotypes: a, comparison to *Col*; b, comparison to *ago4-6 ago9-3* F_2_; c, comparison to *Col*; d, comparison to *ago9-3*. ** *P* < 0.01, *** *P* < 0.001. (C) Whole-mount immunolocalizations showing the expression of AGO6 in wild-type and mutant premeiotic ovules. Alexa 488 fluorescence (green) denotes the localization of the antibody raised against AGO6; samples were counterstained with propidium iodide (red). Bar, 15 µm.

To explore if the compensatory mechanism triggered by the simultaneous loss of function of *AGO4* and *AGO9* could be related to the activity of other members of the *AGO4* clade, we conducted qPCR to determine the expression of *AGO6* and *AGO8* in developing gynoecia harboring premeiotic ovules of single *ago4-6*, *ago9-3*, and double homozygous *ago4-6 ago9-3* individuals (Figure 4, A and B). In *ago4-6*, the expression of *AGO6* was equivalent to wild type (*Col*); however, the expression of *AGO6* was significantly increased in *ago9-3* (Figure 4A), indicating a differential response in the expression of *AGO6* to the absence of *AGO4* or *AGO9* functional activity. Despite being overexpressed in *ago9-3*, premeiotic gynoecia of the double mutant *ago4-6 ago9-3* exhibited normal *AGO6* expression, indicating that loss of function of *AGO4* in the *ago9-3* background negatively regulates *AGO6* activity. In contrast to *AGO6*, the expression of *AGO8* was not affected in *ago4-6* and *ago9-3* single mutants (Figure 4B), but was significantly increased in premeiotic gynoecia of *ago4-6 ago9-3* individuals (Figure 4B). These results indicate that interactions between *AGO4*-clade members are reflected at the level of transcriptional gene activity, suggesting that robust redundant mechanisms among members of the *AGO4* clade contribute to restrict gametophytic cell fate in the premeiotic ovule. Increased expression of *AGO8* in ovules lacking *AGO4* and *AGO9* activity could contribute to explain the compensatory phenotypic effect exhibited by premeiotic ovules.

To determine if the qPCR experiments could reflect the levels of protein expression in the premeiotic ovule, we performed whole-mount immunolocalization in wild-type *Col*, *ago4-6*, *ago9-3*, and double mutant *ago9-3 ago4-6* ovules; using an antibody raised against AGO6 (Figure 4, C–F; Havecker *et al.* 2010). In wild-type ovules, AGO6 was localized in the cytoplasm and sometimes the nucleus of most sporophytic cells of the premeiotic primordium, including the premeiotic MMC (Figure 4D). The localization and level of AGO6 expression was similar to wild type in premeiotic ovules of *ago4-6* and *ago4-6 ago9-3* individuals (Figure 4E), a result in agreement with our previous qPCR assay. At the onset of integumentary initiation, a region of preferential AGO6 expression was identified in the dorsal region of the primordium in both genetic backgrounds (Figure 4D); suggesting that the absence of AGO4 activity might cause subtle differences in the pattern of AGO6 localization, but no differences in the level of AGO6 protein expression. By contrast, AGO6 was abundantly localized throughout the ovule primordium of *ago9-3* plants, including L1 cells of the apical region and the MMC (Figure 4F). These results show that overexpression of AGO6 in *ago9-3* ovules is reflected at the protein level, suggesting that the genetic interactions that control premeiotic gametophytic specification imply dosage effects at the protein level among AGO4-clade members.

## Discussion

### All genes of the *AGO4* clade play an important role during the somatic-to-reproductive transition in the ovule

*ARGONAUTE* genes have been described as fundamental factors controlling specific aspects of germline development in yeast, *Drosophila*, *Caenorhabditis elegans*, mammals, and plants (Lin and Spradling 1997; Kennerdell *et al.* 2002; Yigit *et al.* 2006; Nonomura *et al.* 2007; Olmedo-Monfil *et al.* 2010). Here we provide genetic evidence indicating that all members of the *AGO4*-clade contribute to restrict the number of gametophytic precursors in premeiotic ovules of *Arabidopsis*. Distinct mutations in members of the *AGO4* clade result in variable phenotypic frequencies, suggesting that these genes hierarchically contribute to this restriction. Whereas *AGO4* and *AGO9* appear to play a prevalent role in the mechanism that impedes the differentiation of multiple MMCs, mutations in *AGO6* and *AGO8* show equivalent phenotypic effects, albeit at lower frequencies; suggesting a less determinant redundant function. The role of *AGO6* in gametophytic precursor specification is unexpected as the presence of its messenger RNA is not detected in nucellus or MMCs according to previously published data (Schmidt *et al.* 2011). Although *AGO8* was previously reported as a pseudogene (Takeda *et al.* 2008), the *ago8-1* insertional allele exhibits a frequency of ectopic configurations significantly higher than wild type. This same phenotype has been also found in *ago8-2*, suggesting a functional activity necessary for gametophytic precursor specification. By contrast, and despite harboring severe vegetative defects that could precede pleiotropic abnormalities during female reproductive development, homozygous *ago1-37* and *ago5-1* individuals did not show defects in meiosis or gametophytic precursor differentiation, indicating that the canonical microRNA-dependent pathway is not essential for megasporogenesis, and that premeiotic ovules of individuals defective in *AGO5* are also indistinguishable from wild type (Tucker *et al.* 2012); confirming that MMC specification is not dependent on microRNA function but strictly controlled by members of the AGO4 clade.

Since *AGO4*, *AGO6*, and *AGO9* bind heterochromatic sRNAs through the RdDM pathway, our results reinforce the important participation of epigenetic processes in regulating gametophytic precursor specification in the ovule (Zilberman *et al.* 2003; Zheng *et al.* 2007; Duran-Figueroa and Vielle-Calzada 2010; Olmedo-Monfil *et al.* 2010; Eun *et al.* 2011; Duan *et al.* 2014). Our results also show that mutations in any of the *AGO4*-clade genes, including *AGO4*, are dominant over wild-type alleles for defects affecting gametophytic cell specification in the ovule. This type of inheritance had already been reported for *ago9-2* and *ago9-3* (Olmedo-Monfil *et al.* 2010), suggesting that a dosage-dependent mechanism acting nonautonomously during megasporogenesis is responsible for the mutant phenotype in members of the *AGO4* clade; a model for the mode of action has been proposed elsewhere (Armenta-Medina *et al.* 2011) . A recent report showed that the MMC is marked by a reduction of heterochromatin content compared to its surrounding nucellar cells (She *et al.* 2013). A similar depletion of heterochromatic elements is observed in the ectopic configurations of *ago9*, *sgs3*, and *rdr6* individuals (She *et al.* 2013). In addition, genome-wide studies have proven that loss of RdDM components such as *AGO4* and *AGO6* results in changes of chromatin integrity (Huettel *et al.* 2007; Pikaard *et al.* 2008; Law and Jacobsen 2010; Zhang and Zhu 2012; Stroud *et al.* 2013). A direct role of AGO proteins in chromatin modification has been also described in *C. elegans*, where HRDE-1 directs trimethylation of histone H3 at Lysine 9 (Maine and Kimble 1993; Nishiwaki and Miwa 1998; Buckley *et al.* 2012). Furthermore, *Ago-1* of *Drosophila* plays a crucial role in heterochromatin formation (Pushpavalli *et al.* 2012). Taken together, these results support the hypothesis proposing that members of the *AGO4* clade are required for maintaining the chromatin configuration of nucellar cells in *Arabidopsis* in a dosage-dependent manner. The disruption of any of these genes during the somatic-to-reproductive transition would confer a novel chromatin status that might lead to ectopic differentiation of gametophytic precursors.

### Complex interactions between *AGO4*-clade members

Although genetic interactions between *AGO* members have been addressed by comparing the nature and abundance of their interacting sRNAs as well as the consequence of their functional loss for genomic DNA methylation (Zheng *et al.* 2007; Havecker *et al.* 2010; McCue *et al.* 2015), little emphasis has been given to their possible developmental role, mainly due to a lack of obvious mutant phenotypes during vegetative growth. Here we show that *AGO4* and *AGO9* genetically interact during the somatic-to-reproductive transition in the ovule. With the exception of double heterozygous individuals, all genotypic combinations of alleles simultaneously affecting *AGO4* and *AGO9* exhibit nonadditive effects during megasporogenesis. Interestingly, the complete loss of function of these genes can result in the absence of a mutant phenotype, as compared to wild-type plants; however, our results also suggest that suppression of this mutant phenotype is dependent on the *ago4* allelic variants tested and their genetic backgrounds, likely contributing to the compensatory phenomenon revealed by genetic interactions among members of the *AGO4* clade. Recent reports showed that different ecotypes of *Arabidopsis* exhibit equivalent phenotypes at variable frequencies, and that interecotypic hybridization exacerbates the frequency of supernumerary gametophytic precursors, suggesting that multiple loci control cell specification at the onset of female meiosis (Rodríguez-Leal *et al.* 2015).

Our qPCR experiments show that *AGO8* is overexpressed in homozygous F_2_ *ago4 ago9* ovules, but not in ovules of *ago4* or *ago9* single mutants, suggesting that the mitigation of the abnormal phenotype in the double homozygous mutant background could be influenced by the activity *AGO8*. Contrary to *AGO8*, the expression of *AGO6* is increased in *ago9-3* but not in in *ago4-6*, nor in double homozygous *ago4 ago9* individuals. These contrasting effects in *AGO6* and *AGO8* activity suggest that, despite their common contribution to restricting gametophytic precursor specification at the onset of meiosis, each gene is differentially regulated in response to loss of function of *AGO4* or *AGO9*. The recovery of normal expression levels for *AGO6* in the double homozygous *ago4 ago9* but not in single *ago9-3* individuals indicates that *AGO4* influences *AGO6* expression only in the absence of *AGO9* activity. Our overall results suggest that *AGO4*-clade gene members coordinately act to restrict the ectopic formation of gametophytic precursors in the ovule. Because *AGO4* and *AGO9* are importantly required for silencing repetitive elements such as TEs in the germline (Zilberman *et al.* 2003; Duran-Figueroa and Vielle-Calzada 2010), and AGO9 has been implicated in male meiosis and somatic DNA repair (Oliver *et al.* 2014), the compensatory effect revealed by interactions among AGO4-clade members is likely to be extended to a broader developmental context. In addition, genome-wide DNA methylation analysis has revealed nonredundant interactions between *AGO4* and *AGO6* in most of their target loci, as both are required to confer a wild-type DNA methylation status (Duan *et al.* 2014). Interestingly, methylation is not completely suppressed at target loci in *ago4 ago6* mutants, suggesting that additional AGOs are also required to maintain the global methylation pattern (Duan *et al.* 2014).

The pattern of protein localization of AGO4, AGO6, and AGO9 is in agreement with the evidence indicating they play redundant functions during early ovule formation. Whereas AGO4 is ubiquitously expressed throughout development and localized in both nucleus and cytoplasm (Li *et al.* 2006; Ye *et al.* 2012), AGO6 is reported to localize in the cytoplasm of foliar parenchyma cells (Zheng *et al.* 2007; McCue *et al.* 2015). AGO9 is preferentially expressed in the L1 layer of the premeiotic ovule primordium, and in the cytoplasm of nucellar cells (Olmedo-Monfil *et al.* 2010; Escobar-Guzmán *et al.* 2015). Recent evidence indicates that AGO4 and AGO6 differ in their subnuclear colocalization as compared to the RNA polymerases required for RdDM (McCue *et al.* 2015); whereas Pol V and AGO4 are colocalized in perinuclear foci, Pol II and AGO6 are absent (McCue *et al.* 2014).

### Parental genotypes influence the somatic-to-reproductive transition during consecutive generations

A significantly different frequency of ectopic configurations was obtained in double heterozygous *ago4/+ ago9/+* F_1_ and F_2_ individuals, indicating that epigenetic factors are influencing the number of ectopic configurations that differentiate at each generation. Epigenetic variability between parental lines can cause additive effects for developmental traits scored in the progeny (Groszmann *et al.* 2013; Groszmann *et al.* 2014; Escobar-Guzmán *et al.* 2015). Despite the large proportion of potential RdDM target loci shared by both genes, there is also evidence suggesting specific epigenetic regulation by one or the other (Havecker *et al.* 2010), suggesting potential epi-allelism for each mutant. Strikingly, segregant *ago4/+ ago9/+* F_3_ individuals showed frequencies equivalent to *ago4/+ ago9/+* F_2_ plants, suggesting that double heterozygous progeny from outcrossed plants behave differently than heterozygous progeny of self-fertilized individuals.

Double homozygous *ago4 ago9* individuals derived from self-pollination of double heterozygous plants showed frequencies of ectopic configurations equivalent to wild type. In contrast, *ago4 ago9* individuals derived from self-pollination of a double homozygous plant showed an abnormal frequency of ectopic configurations, revealing an influence of the parental genotype on the capacity for restricting ectopic differentiation of female gamete precursors. The frequency of ectopic configurations in double homozygous *ago4 ago9* individuals increased progressively throughout consecutive generations, indicating that the compensatory effect is progressively diminished. These results suggest that *AGO4* and *AGO9* are necessary for maintaining the epigenetic marks that ensure the restriction of gametogenic commitment to a single cell, over consecutive generations. There is growing evidence that epigenetic modifications occurring within the plant germline in one generation can be stably inherited at subsequent generations (Iwasaki and Paszkowski 2014). For example, components of the RdDM pathway have been implicated in preventing transgenerational accumulation of some Ty1/Copia-like retrotransposons in plants affected by abiotic stress in *Arabidopsis* (Ito *et al.* 2011); and in *C. elegans*, proteins such as HRDE-1 are required for transmitting the RNA-interference silencing signal to subsequent generations (Buckley *et al.* 2012). Our results indicate that *AGO4* and *AGO9* are required to establish the transgenerational epigenetic information that is necessary to restrict gametophytic fate in the ovule, confirming that members of the *AGO4* clade cooperatively participate in preventing the abnormal specification of multiple premeiotic gametophytic precursors during early ovule development.

### Conclusions

We show a surprising degree of cooperative interaction among gene members of the *AGO4* clade during meiosis and megaspore formation in *Arabidopsis*. Our study reveals unforeseen levels of epigenetic control acting to canalize a developmental process that is essential for sexual plant reproduction in flowering plants.

## References

[bib1] Bajon C. Horlow C. Motamayor J. C. Sauvanet A. Robert D., 1999 Megasporogenesis in Arabidopsos thaliana L.: an ultrastructural study. Sex. Plant Reprod. 12: 99–109.

[bib2] Bendel-Stenzel M. Anderson R. Heasman J. Wylie C., 1998 The origin and migration of primordial germ cells in the mouse. Semin. Cell Dev. Biol. 9: 393–400. 9813186 10.1006/scdb.1998.0204

[bib3] Brennecke J. Aravin A. A. Stark A. Dus M. Kellis M., 2007 Discrete small RNA-generating loci as master regulators of transposon activity in Drosophila. Cell 128: 1089–1103. 17346786 10.1016/j.cell.2007.01.043

[bib4] Brennecke J. Malone C. D. Aravin A. A. Sachidanandam R. Stark A., 2008 An epigenetic role for maternally inherited piRNAs in transposon silencing. Science 322: 1387–1392. 19039138 10.1126/science.1165171 PMC2805124

[bib5] Buckley B. A. Burkhart K. B. Gu S. G. Spracklin G. Kershner A., 2012 A nuclear argonaute promotes multigenerational epigenetic inheritance and germline immortality. Nature 489: 447–451. 22810588 10.1038/nature11352 PMC3509936

[bib6] Czechowski T. Bari R. P. Stitt M. Scheible W. R. Udvardi M. K., 2004 Real-time RT-PCR profiling of over 1400 Arabidopsis transcription factors: unprecedented sensitivity reveals novel root- and shoot-specific genes. Plant J. 38: 366–379. 15078338 10.1111/j.1365-313X.2004.02051.x

[bib7] Donoughe S. Nakamura T. Ewen-Campen B. Green D. A. II Henderson L., 2014 BMP signaling is required for the generation of primordial germ cells in an insect. Proc. Natl. Acad. Sci. USA 111: 4133–4138. 24591634 10.1073/pnas.1400525111 PMC3964053

[bib8] Drews G. N. Yadegari R., 2002 Development and function of the angiosperm female gametophyte. Annu. Rev. Genet. 36: 99–124. 12429688 10.1146/annurev.genet.36.040102.131941

[bib9] Duan C. G. Zhang H. Tang K. Zhu X. Qian W., 2014 Specific but interdependent functions for Arabidopsis AGO4 and AGO6 in RNA-directed DNA methylation. EMBO J. 34: 581–592. 25527293 10.15252/embj.201489453 PMC4365029

[bib10] Duran-Figueroa N. Vielle-Calzada J. P., 2010 ARGONAUTE9-dependent silencing of transposable elements in pericentromeric regions of Arabidopsis. Plant Signal. Behav. 5: 1476–1479. 21057207 10.4161/psb.5.11.13548 PMC3115260

[bib11] Escobar-Guzmán R. Rodríguez-Leal D. Vielle-Calzada J.-P. Ronceret A., 2015 Whole-mount immunolocalization to study female meiosis in Arabidopsis. Nat. Protoc. 10: 1535–1542. 26357009 10.1038/nprot.2015.098

[bib12] Eun C. Lorkovic Z. J. Naumann U. Long Q. Havecker E. R., 2011 AGO6 functions in RNA-mediated transcriptional gene silencing in shoot and root meristems in Arabidopsis thaliana. PLoS One 6: e25730. 21998686 10.1371/journal.pone.0025730 PMC3187791

[bib13] Groszmann M. Greaves I. K. Fujimoto R. Peacock W. J. Dennis E. S., 2013 The role of epigenetics in hybrid vigour. Trends Genet. 29: 684–690. 23953922 10.1016/j.tig.2013.07.004

[bib14] Groszmann M. Gonzalez-Bayon R. Greaves I. K. Wang L. Huen A. K., 2014 Intraspecific Arabidopsis hybrids show different patterns of heterosis despite the close relatedness of the parental genomes. Plant Physiol. 166: 265–280. 25073707 10.1104/pp.114.243998 PMC4149712

[bib15] Hajkova P. Erhardt S. Lane N. Haaf T. El-Maarri O., 2002 Epigenetic reprogramming in mouse primordial germ cells. Mech. Dev. 117: 15–23. 12204247 10.1016/s0925-4773(02)00181-8

[bib16] Havecker E. R. Wallbridge L. M. Hardcastle T. J. Bush M. S. Kelly K. A., 2010 The Arabidopsis RNA-directed DNA methylation argonautes functionally diverge based on their expression and interaction with target loci. Plant Cell 22: 321–334. 20173091 10.1105/tpc.109.072199 PMC2845420

[bib17] Havecker E. R. Wallbridge L. M. Fedito P. Hardcastle T. J. Baulcombe D. C., 2012 Metastable differentially methylated regions within Arabidopsis inbred populations are associated with modified expression of non-coding transcripts. PLoS One 7: e45242. 23028873 10.1371/journal.pone.0045242 PMC3447930

[bib18] Huettel B. Kanno T. Daxinger L. Bucher E. van der Winden J., 2007 RNA-directed DNA methylation mediated by DRD1 and Pol IVb: a versatile pathway for transcriptional gene silencing in plants. Biochim. Biophys. Acta 1769: 358–374. 17449119 10.1016/j.bbaexp.2007.03.001

[bib19] Ito H. Gaubert H. Bucher E. Mirouze M. Vaillant I., 2011 An siRNA pathway prevents transgenerational retrotransposition in plants subjected to stress. Nature 472: 115–119. 21399627 10.1038/nature09861

[bib20] Iwasaki M. Paszkowski J., 2014 Epigenetic memory in plants. EMBO J. 33: 1987–1998. 25104823 10.15252/embj.201488883 PMC4195768

[bib21] Johannes F. Colome-Tatche M., 2011 Quantitative epigenetics through epigenomic perturbation of isogenic lines. Genetics 188: 215–227. 21385727 10.1534/genetics.111.127118 PMC3120148

[bib22] Kelly W. G., 2014 Transgenerational epigenetics in the germline cycle of Caenorhabditis elegans. Epigenetics Chromatin 7: 6. 24678826 10.1186/1756-8935-7-6 PMC3973826

[bib23] Kennerdell J. R. Yamaguchi S. Carthew R. W., 2002 RNAi is activated during Drosophila oocyte maturation in a manner dependent on aubergine and spindle-E. Genes Dev. 16: 1884–1889. 12154120 10.1101/gad.990802 PMC186417

[bib24] Law J. A. Jacobsen S. E., 2010 Establishing, maintaining and modifying DNA methylation patterns in plants and animals. Nat. Rev. Genet. 11: 204–220. 20142834 10.1038/nrg2719 PMC3034103

[bib25] Leitch H. G. McEwen K. R. Turp A. Encheva V. Carroll T., 2013 Naive pluripotency is associated with global DNA hypomethylation. Nat. Struct. Mol. Biol. 20: 311–316. 23416945 10.1038/nsmb.2510 PMC3591483

[bib26] Li C. F. Pontes O. El-Shami M. Henderson I. R. Bernatavichute Y. V., 2006 An ARGONAUTE4-containing nuclear processing center colocalized with Cajal bodies in Arabidopsis thaliana. Cell 126: 93–106. 16839879 10.1016/j.cell.2006.05.032

[bib27] Lieber D. Lora J. Schrempp S. Lenhard M. Laux T., 2011 Arabidopsis WIH1 and WIH2 genes act in the transition from somatic to reproductive cell fate. Curr. Biol. 21: 1009–1017. 21658947 10.1016/j.cub.2011.05.015

[bib28] Lin H. Spradling A. C., 1997 A novel group of pumilio mutations affects the asymmetric division of germline stem cells in the Drosophila ovary. Development 124: 2463–2476. 9199372 10.1242/dev.124.12.2463

[bib29] Loriot A. Boon T. De Smet C., 2003 Five new human cancer-germline genes identified among 12 genes expressed in spermatogonia. Int. J. Cancer 105: 371–376. 12704671 10.1002/ijc.11104

[bib30] Maine E. M. Kimble J., 1993 Suppressors of glp-1, a gene required for cell communication during development in Caenorhabditis elegans, define a set of interacting genes. Genetics 135: 1011–1022. 8307319 10.1093/genetics/135.4.1011 PMC1205734

[bib31] Mari-Ordonez A. Marchais A. Etcheverry M. Martin A. Colot V., 2013 Reconstructing de novo silencing of an active plant retrotransposon. Nat. Genet. 45: 1029–1039. 23852169 10.1038/ng.2703

[bib32] Matzke M. A. Mosher R. A., 2014 RNA-directed DNA methylation: an epigenetic pathway of increasing complexity. Nat. Rev. Genet. 15: 394–408. 24805120 10.1038/nrg3683

[bib33] McCue A. D. Panda K. Nuthikattu S. Choudury S. G. Thomas E. N., 2015 ARGONAUTE 6 bridges transposable element mRNA-derived siRNAs to the establishment of DNA methylation. EMBO J. 34: 20–35. 25388951 10.15252/embj.201489499 PMC4291478

[bib34] Nishiwaki K. Miwa J., 1998 Mutations in genes encoding extracellular matrix proteins suppress the emb-5 gastrulation defect in Caenorhabditis elegans. Mol. Gen. Genet. 259: 2–12. 9738874 10.1007/s004380050782

[bib35] Nonomura K. Miyoshi K. Eiguchi M. Suzuki T. Miyao A., 2003 The MSP1 gene is necessary to restrict the number of cells entering into male and female sporogenesis and to initiate anther wall formation in rice. Plant Cell 15: 1728–1739. 12897248 10.1105/tpc.012401 PMC167165

[bib36] Nonomura K. Morohoshi A. Nakano M. Eiguchi M. Miyao A., 2007 A germ cell specific gene of the argonaute family is essential for the progression of premeiotic mitosis and meiosis during sporogenesis in rice. Plant Cell 19: 2583–2594. 17675402 10.1105/tpc.107.053199 PMC2002623

[bib37] Olmedo-Monfil V. Duran-Figueroa N. Arteaga-Vazquez M. Demesa-Arevalo E. Autran D., 2010 Control of female gamete formation by a small RNA pathway in Arabidopsis. Nature 464: 628–632. 20208518 10.1038/nature08828 PMC4613780

[bib38] Pikaard C. S. Haag J. R. Ream T. Wierzbicki A. T., 2008 Roles of RNA polymerase IV in gene silencing. Trends Plant Sci. 13: 390–397. 18514566 10.1016/j.tplants.2008.04.008 PMC2679257

[bib39] Pushpavalli S. N. Bag I. Pal-Bhadra M. Bhadra U., 2012 Drosophila Argonaute-1 is critical for transcriptional cosuppression and heterochromatin formation. Chromosome Res. 20: 333–351. 22476395 10.1007/s10577-012-9279-y PMC3323821

[bib40] Reinders J. Paszkowski J., 2009 Unlocking the Arabidopsis epigenome. Epigenetics 4: 557–563. 19934651 10.4161/epi.4.8.10347

[bib41] Reiser L. Fischer R. L., 1993 The ovule and the embryo sac. Plant Cell 5: 1291–1301. 12271029 10.1105/tpc.5.10.1291 PMC160362

[bib42] Robinson-Beers K. Pruitt R. E. Gasser C. S., 1992 Ovule development in wild-type Arabidopsis and two female-sterile mutants. Plant Cell 4: 1237–1249. 12297633 10.1105/tpc.4.10.1237 PMC160211

[bib67] Rodríguez-Leal D. León-Martínez G. Abad-Vivero U. Vielle-Calzada J. P., 2015 Natural variation in epigenetic pathways affects the specification of female gamete precursors in Arabidopsis. Plant Cell 4: 1034–1045. 10.1105/tpc.114.133009 PMC4558685 25829442

[bib43] Rowley M. J. Avrutsky M. I. Sifuentes C. J. Pereira L. Wierzbicki A. T., 2011 Independent chromatin binding of ARGONAUTE4 and SPT5L/KTF1 mediates transcriptional gene silencing. PLoS Genet. 7: e1002120. 21738482 10.1371/journal.pgen.1002120 PMC3111484

[bib44] Schmidt A. Wuest S. E. Vijverberg K. Baroux C. Kleen D., 2011 Transcriptome analysis of the Arabidopsis megaspore mother cell uncovers the importance of RNA helicases for plant germline development. PLoS Biol. 9: e1001155. 21949639 10.1371/journal.pbio.1001155 PMC3176755

[bib45] Schneitz K. Hülskamp M. Pruitt R. E., 1995 Wild-type ovule development in Arabidopsis thaliana: a light microscope study of cleared whole-mount tissue. Plant J. 7: 731–749.

[bib46] Seki Y. Hayashi K. Itoh K. Mizugaki M. Saitou M., 2005 Extensive and orderly reprogramming of genome-wide chromatin modifications associated with specification and early development of germ cells in mice. Dev. Biol. 278: 440–458. 15680362 10.1016/j.ydbio.2004.11.025

[bib47] She W. Grimanelli D. Rutowicz K. Whitehead M. W. Puzio M., 2013 Chromatin reprogramming during the somatic-to-reproductive cell fate transition in plants. Development 140: 4008–4019. 24004947 10.1242/dev.095034

[bib48] Sheridan W. F. Golubeva E. A. Abrhamova L. I. Golubovskaya I. N., 1999 The mac1 mutation alters the developmental fate of the hypodermal cells and their cellular progeny in the maize anther. Genetics 153: 933–941. 10511568 10.1093/genetics/153.2.933 PMC1460776

[bib49] Simon S. A. Meyers B. C., 2010 Small RNA-mediated epigenetic modifications in plants. Curr. Opin. Plant Biol. 14: 148–155. 21159545 10.1016/j.pbi.2010.11.007

[bib50] Strickler S. R. Tantikanjana T. Nasrallah J. B., 2013 Regulation of the S-locus receptor kinase and self-incompatibility in Arabidopsis thaliana. G3 (Bethesda) 3: 315–322. 23390607 10.1534/g3.112.004879 PMC3564991

[bib51] Stroud H. Greenberg M. V. Feng S. Bernatavichute Y. V. Jacobsen S. E., 2013 Comprehensive analysis of silencing mutants reveals complex regulation of the Arabidopsis methylome. Cell 152: 352–364. 23313553 10.1016/j.cell.2012.10.054 PMC3597350

[bib52] Takeda A. Iwasaki S. Watanabe T. Utsumi M. Watanabe Y., 2008 The mechanism selecting the guide strand from small RNA duplexes is different among argonaute proteins. Plant Cell Physiol. 49: 493–500. 18344228 10.1093/pcp/pcn043

[bib53] Tucker M. R. Okada T. Hu Y. Scholefield A. Taylor J. M., 2012 Somatic small RNA pathways promote the mitotic events of megagametogenesis during female reproductive development in Arabidopsis. Development 139: 1399–1404. 22399683 10.1242/dev.075390

[bib54] Vaucheret H., 2008 Plant argonautes. Trends Plant Sci. 13: 350–358. 18508405 10.1016/j.tplants.2008.04.007

[bib55] Yadegari R. Drews G. N., 2004 Female gametophyte development. Plant Cell 16: S133–S141. 15075395 10.1105/tpc.018192 PMC2643389

[bib56] Yang L. Huang W. Wang H. Cai R. Xu Y., 2006 Characterizations of a hypomorphic argonaute1 mutant reveal novel AGO1 functions in Arabidopsis lateral organ development. Plant Mol. Biol. 61: 63–78. 16786292 10.1007/s11103-005-5992-7

[bib57] Yang W. C. Ye D. Xu J. Sundaresan V., 1999 The SPOROCYTELESS gene of Arabidopsis is required for initiation of sporogenesis and encodes a novel nuclear protein. Genes Dev. 13: 2108–2117. 10465788 10.1101/gad.13.16.2108 PMC316961

[bib58] Ye R. Wang W. Iki T. Liu C. Wu Y., 2012 Cytoplasmic assembly and selective nuclear import of Arabidopsis Argonaute4/siRNA complexes. Mol. Cell 46: 859–870. 22608924 10.1016/j.molcel.2012.04.013

[bib59] Yigit E. Batista P. J. Bei Y. Pang K. M. Chen C. C., 2006 Analysis of the C. elegans Argonaute family reveals that distinct Argonautes act sequentially during RNAi. Cell 127: 747–757. 17110334 10.1016/j.cell.2006.09.033

[bib60] Zhai L. Sun W. Zhang K. Jia H. Liu L., 2014 Identification and characterization of Argonaute gene family and meiosis-enriched Argonaute during sporogenesis in maize. J. Integr. Plant Biol. 56: 1042–1052. 24735215 10.1111/jipb.12205

[bib61] Zhang H. Zhu J. K., 2012 Seeing the forest for the trees: a wide perspective on RNA-directed DNA methylation. Genes Dev. 26: 1769–1773. 22895250 10.1101/gad.200410.112 PMC3426756

[bib62] Zheng Q. Rowley M. J. Bohmdorfer G. Sandhu D. Gregory B. D., 2013 RNA polymerase V targets transcriptional silencing components to promoters of protein-coding genes. Plant J. 73: 179–189. 23013441 10.1111/tpj.12034 PMC5096367

[bib63] Zheng X. Zhu J. Kapoor A. Zhu J. K., 2007 Role of Arabidopsis AGO6 in siRNA accumulation, DNA methylation and transcriptional gene silencing. EMBO J. 26: 1691–1701. 17332757 10.1038/sj.emboj.7601603 PMC1829372

[bib64] Zhong X. Du J. Hale C. J. Gallego-Bartolome J. Feng S., 2014 Molecular mechanism of action of plant DRM de novo DNA methyltransferases. Cell 157: 1050–1060. 24855943 10.1016/j.cell.2014.03.056 PMC4123750

[bib65] Zilberman D. Cao X. Jacobsen S. E., 2003 ARGONAUTE4 control of locus-specific siRNA accumulation and DNA and histone methylation. Science 299: 716–719. 12522258 10.1126/science.1079695

[bib66] Zilberman D. Cao X. Johansen L. K. Xie Z. Carrington J. C., 2004 Role of Arabidopsis ARGONAUTE4 in RNA-directed DNA methylation triggered by inverted repeats. Curr. Biol. 14: 1214–1220. 15242620 10.1016/j.cub.2004.06.055

